# Hypothermic Preconditioning of Human Cortical Neurons Requires Proteostatic Priming

**DOI:** 10.1016/j.ebiom.2015.04.004

**Published:** 2015-04-11

**Authors:** Nina Marie Rzechorzek, Peter Connick, Rickie Patani, Bhuvaneish Thangaraj Selvaraj, Siddharthan Chandran

**Affiliations:** a Centre for Clinical Brain Sciences, University of Edinburgh, Midlothian EH16 4SB, United Kingdom; b MRC Centre for Regenerative Medicine, University of Edinburgh, Midlothian EH16 4SB, United Kingdom; c The Anne Rowling Regenerative Neurology Clinic, University of Edinburgh, Midlothian EH16 4SB, United Kingdom; d Department of Molecular Neuroscience, UCL Institute of Neurology, Queen Square, London WC1N 3BG, United Kingdom

**Keywords:** Endoplasmic reticulum stress, Unfolded protein response, Hypothermia, Preconditioning, Proteostasis, Neuroprotection

## Abstract

Hypothermia is potently neuroprotective but poor mechanistic understanding has restricted its clinical use. Rodent studies indicate that hypothermia can elicit preconditioning, wherein a subtoxic cellular stress confers resistance to an otherwise lethal injury. The molecular basis of this preconditioning remains obscure. Here we explore molecular effects of cooling using functional cortical neurons differentiated from human pluripotent stem cells (hCNs). Mild-to-moderate hypothermia (28–32 °C) induces cold-shock protein expression and mild endoplasmic reticulum (ER) stress in hCNs, with full activation of the unfolded protein response (UPR). Chemical block of a principal UPR pathway mitigates the protective effect of cooling against oxidative stress, whilst pre-cooling neurons abrogates the toxic injury produced by the ER stressor tunicamycin. Cold-stress thus preconditions neurons by upregulating adaptive chaperone-driven pathways of the UPR in a manner that precipitates ER-hormesis. Our findings establish a novel arm of neurocryobiology that could reveal multiple therapeutic targets for acute and chronic neuronal injury.

## Highlights

• Clinically-relevant cooling induces archetypal cold-shock and mild endoplasmic reticulum (ER) stress in human neurons.
• Hypothermic neuronal ER-stress elicits an adaptive unfolded protein response (UPR) with ER-hormesis.
• Hypothermic preconditioning of the ER provides cross-tolerance to oxidative neuronal injury and requires an intact UPR.

## 1 Introduction

Therapeutic cooling offers robust protection against ischaemic brain damage, but its practical challenges and risks have limited its application to specific patient groups (Choi et al., 2012, Yenari and Han, 2012). Advancing our insight into cooling-induced neuroprotection at the cellular level could provide new molecular targets to bypass the need for cooling — whilst expanding its therapeutic potential. Preconditioning describes the tolerance achieved against an intensively toxic insult by subjecting cells or tissue to a sublethal stress (Stetler et al., 2014). Neuronal preconditioning can be effected by many and varied stimuli, including hypothermia (Dirnagl et al., 2003, Yuan et al., 2004, Stetler et al., 2014). In rodents, this cooling-induced tolerance requires de novo protein synthesis (Nishio et al., 2000) — a fundamental arm of the cold-shock response (Fujita, 1999), for which data in human neurons is lacking. Depending on the depth of cooling, this response leads to cell-cycle arrest with shut-down of transcription and translation (Yenari and Han, 2012). Simultaneously, a subset of highly conserved ‘cold-inducible’ RNA chaperones including RNA binding motif 3 (RBM3) and cold-inducible RNA binding protein (CIRBP) is rapidly upregulated (Lleonart, 2010). These ‘cold-shock’ proteins mediate important survival functions including facilitated translation of essential mRNAs and suppression of apoptosis (Lleonart, 2010, Saito et al., 2010).

Aside from induction of cold-shock proteins however, little is known of other fundamental cellular stress pathways in relation to cooling and their potential relevance to hypothermic preconditioning (Hofman et al., 2012, van der Harg et al., 2014). Hypothermia can induce protein unfolding and disrupt the cell secretory pathway (Saraste et al., 1986, Liu et al., 1994, Fujita, 1999), both of which would result in endoplasmic reticulum (ER) stress (Kim et al., 2008). However, mammalian cell lines have produced conflicting data regarding the ability of cooling to trigger ER stress and downstream events coordinated by the unfolded protein response (UPR) (Hofman et al., 2012, van der Harg et al., 2014). Although this may relate to the variable depths of hypothermia studied, it likely also reflects the resistance of immortal cell types to physiological stress (Abdel Malek et al., 2015, Cerezo et al., 2015). Furthermore, the ER-UPR cascade as a whole has never been explored at clinically-relevant hypothermic temperatures. Potentially, such a moderate level of cold-stress might bring about an adaptive proteostatic response in post-mitotic neurons (Mendes et al., 2009, Fouillet et al., 2012). Here we test this hypothesis by characterizing the cold-shock response to protective hypothermia in functional cortical neurons differentiated from human pluripotent stem cells (hCNs) (Bilican et al., 2014), using this model to explore the molecular basis of hypothermic preconditioning.

## 2 Materials and Methods

### 2.1 Human Brain Tissue

Human post-mortem cortical brain tissue was obtained under full ethical and Institutional Review Board approval of the University of Edinburgh. Adult samples (healthy control, 17 y) were provided by the MRC Edinburgh Brain & Tissue Bank. Foetal samples were procured after elective surgical abortion (gestation 16 w), with full ethics permission of the NHS Lothian Research Ethics Committee (REC 08/S1101/1). Post-mortem samples were included as positive (foetal) and negative (adult) controls for RBM3 and CIRBP expression, which is developmentally-regulated in the human cortex (Miller et al., 2014 and The Allen Institute for Brain Science).

### 2.2 Cell Culture

All culture experiments were performed using hCNs derived from human pluripotent stem cell lines. Two human embryonic stem cell (hES) lines (H9, female, WiCell, Madison, WI and Shef 4, male, UK Stem Cell Bank, designated HES1 and HES2) were obtained under full ethical and Institutional Review Board approval of the University of Edinburgh. One human induced pluripotent stem cell (iPS) line (IPS1, healthy female control) was reprogrammed in-house after obtaining written informed consent and ethics permission (REC/10/S1103/10). hCN differentiation and immunocytochemistry protocols are described in Bilican et al. (2014). Primary antibodies (Abs) included: βIII-tubulin (mouse monoclonal, Sigma), RBM3 (rabbit monoclonal, Abcam) and CIRBP (rabbit polyclonal, Pierce). Cell counts were performed blind to the temperature variable.

### 2.3 Cooling and Multiplexed Injury Assays

Hypothermia was induced at 5 w when > 90% of hCNs are functional (Bilican et al., 2014, Livesey et al., 2014). Identical plates were cultured at 28, 32 or 37 °C to simulate ‘moderate hypothermia’, ‘mild hypothermia’ or ‘normothermia’, respectively (Yenari and Han, 2012). Samples for transcript analysis were lifted at 3 and 24 h, after which additional samples were processed for immunocytochemistry and biochemistry. For oxidative injury experiments, neurons were switched to minimal medium (MiM (Gupta et al., 2013)) containing no antioxidants 12 h prior to temperature shift as above. After 24 h, H_2_O_2_ (diluted in MiM) was applied at 0, 50, 100 or 200 μM via 50% media exchange. Control wells received MiM with vehicle only. After a further 24 h at the respective temperatures, culture media was harvested for the cytotoxicity assay (CytoTox-One™, Promega), and cells lysed for the viability assay (CellTiter-Glo®, Promega). LDH release (cytotoxicity) was read fluorometrically (excitation 560 and emission 590 nm), whilst ATP production (viability) was measured via luminescence (Promega Glomax). Readings were taken in triplicate and averaged for each condition, after subtracting values for MiM only (fluorescence) and no cell control (luminescence). The derived ‘injury ratio’ (cytotoxicity in relative fluorescent units (RFU) divided by viability in relative luminescent units (RLU)) obtained for each well of cells adjusted for any potential inter-well variation in cell number. To evaluate baseline toxicity of tunicamycin (Tm) and protein kinase R (PKR)-like ER kinase (PERK) inhibitor, neurons were switched to MiM 12 h before a 24 h exposure to these compounds at 37 °C followed by multiplexed injury analysis as above.

### 2.4 Quantitative Real-Time PCR (qRT-PCR)

RNA extraction, cDNA synthesis and qRT-PCR were performed as described (Bilican et al., 2014) using primers listed in Supplementary Materials and Methods. Validation of reference target stability in hCNs under hypothermic conditions was determined with a combination of geNorm (qbase +, Biogazelle) and NormFinder (Excel) analysis (Vandesompele et al., 2002). qRT-PCR reactions were performed in triplicate and average target transcript expression was normalized to the geometric mean of eukaryotic translation initiation factor 4A2 (EIF4A2) and glyceraldehyde 3-phosphate dehydrogenase (GAPDH) expression for each sample.

### 2.5 Quantitative Western Analysis

Cell pellets were harvested in ice-cold Tris-buffered saline (TBS) containing protease inhibitors (cOmplete ULTRA, Roche), and where necessary, phosphatase inhibitors (PhosSTOP, Roche). Post-mortem samples were divided into 200–300 mg pieces at 4 °C. Protein was extracted on ice in radioimmunoprecipitation assay (RIPA) buffer (50 mM Tris pH 8, 150 mM NaCl, 1% Triton-X 100, 5 mM ethylenediaminetetraacetic acid (EDTA), 0.5% Na·deoxycholate (w/v), 0.1% sodium dodecyl sulphate (SDS)) containing protease inhibitors (as above, plus 100 μM phenylmethanesulfonylfluoride (PMSF), Fluka BioChemika). Tissue samples required homogenization (Precyllys®24). Lysates were ultracentrifuged (20 min, 50,000 ×*g*, 4 °C, Beckman). RIPA-insoluble pellets were further extracted for 20 min in 2% SDS RIPA followed by repeat ultracentrifugation to isolate nuclear proteins. Protein concentration was measured (BCA assay, Pierce) and samples boiled prior to SDS-polyacrylamide gel electrophoresis (PAGE) (4–20% gradient gels, Thermoscientific). Proteins were transferred onto Immobilon®-FL polyvinylidene fluoride (PVDF) membranes (Millipore) and blocked for 45 min at room temperature (Odyssey™ Blocking Buffer, LI-COR® Biosciences). Membranes were incubated overnight at 4 °C with primary Abs: activating transcription factor 6 (ATF6 at 1:100, mouse monoclonal, Abcam), binding immunoglobulin protein (BiP; also known as glucose-regulated protein, 78 kDa (GRP78) or heat shock 70 kDa protein 5 (HSPA5) at 1:1000, rabbit monoclonal, Abcam), CIRBP (at 1:500, rabbit polyclonal, Proteintech), eukaryotic initiation factor 2α (eIF2α at 1:1000, mouse monoclonal, Abcam), phospho-eIF2α (p-eIF2α at 1:100, rabbit monoclonal, Cell Signalling), PERK (at 1:100, rabbit monoclonal, Cell Signalling), RBM3 (at 1:100, rabbit monoclonal, Abcam), GAPDH (at 1:10,000, mouse monoclonal, Calbiochem) or heterogeneous nuclear ribonucleoprotein (hnRNP) A1 (at 1:1000, mouse monoclonal, Santa Cruz), then probed for 1 h at room temperature with Fluorescent conjugated secondary Abs (IRDye®680RD Goat (polyclonal) Anti-Rabbit IgG (H + L) and IRDye® 800CW Goat (polyclonal) Anti-Mouse IgG (H + L), LI-COR® Biosciences). Blots were exposed for 10 min (at 700 nm and/or 800 nm, LI-COR® Odyssey Fc Dual-Mode Imaging System), with band intensities quantified in Image Studio. Samples for each independently plated batch of cells were run in triplicate and average intensity readings were normalized to their respective loading control expression or total eIF2α expression (for p-eIF2α quantification).

### 2.6 XBP1 Splicing Assay

hCNs were treated for 24 h with hypothermia or Tm (0.3 μg/ml). qRT-PCR was performed as above. Conventional RT-PCR was performed using Quick-Load® Taq 2X Master Mix (New England Biolabs) and a BioRad C1000 Thermal Cycler (annealing temperature 60 °C). GAPDH and c-Myc (MYC) were included for reference and to confirm a stress response respectively. Products were resolved on 2.5% agarose gels.

### 2.7 Media and Supplements

Components were purchased from Invitrogen unless otherwise stated. hCN differentiation medium is described elsewhere (Bilican et al., 2014, Livesey et al., 2014). MiM comprised 90% salt–glucose–glycine solution (Bading et al., 1993) with 10% Minimal Eagle's Medium (+ Earle's, − Glutamine) and 0.5% Penicillin–Streptomycin. Tm (0.3 μg/ml, Sigma) and PERK inhibitor (GSK2606414, 500 nM, Calbiochem) were applied from the start of the temperature shift (they were present at these concentrations throughout the 24 h preconditioning phase and were then diluted by 50% upon the addition of H_2_O_2_ for the 24 h injury phase).

### 2.8 Statistical Analysis

Pairwise correlations were performed by two-tailed Pearson correlation. Remaining analyses were performed using linear mixed models in Stata SE (Version 9.2, Stata Corp, TX, USA) with random effects for intercept by batch, and where necessary, random effects for coefficient by concentration or time. *N* denotes the number of individual cell lines and *n* describes the total number of independently differentiated batches of hCNs used as the statistical *n* for each experiment (the number of independent observations). The number (*n_line_*) of batches derived from each cell line is then stated in parenthesis to show the contribution of each biological entity to the pooled total. Unless otherwise stated, data are presented as standardized point estimates (SPE) + standardized estimated standard error (SESE) after normalizing to control values (normothermic or untreated cells). Asterisks denote significance of the test statistic: *P < 0.05, **P < 0.01, ***P < 0.001, ****P < 0.0005.

## 3 Results

### 3.1 Human Neurons Exhibit an Archetypal Cold-Shock Response

**Fig. 1:**
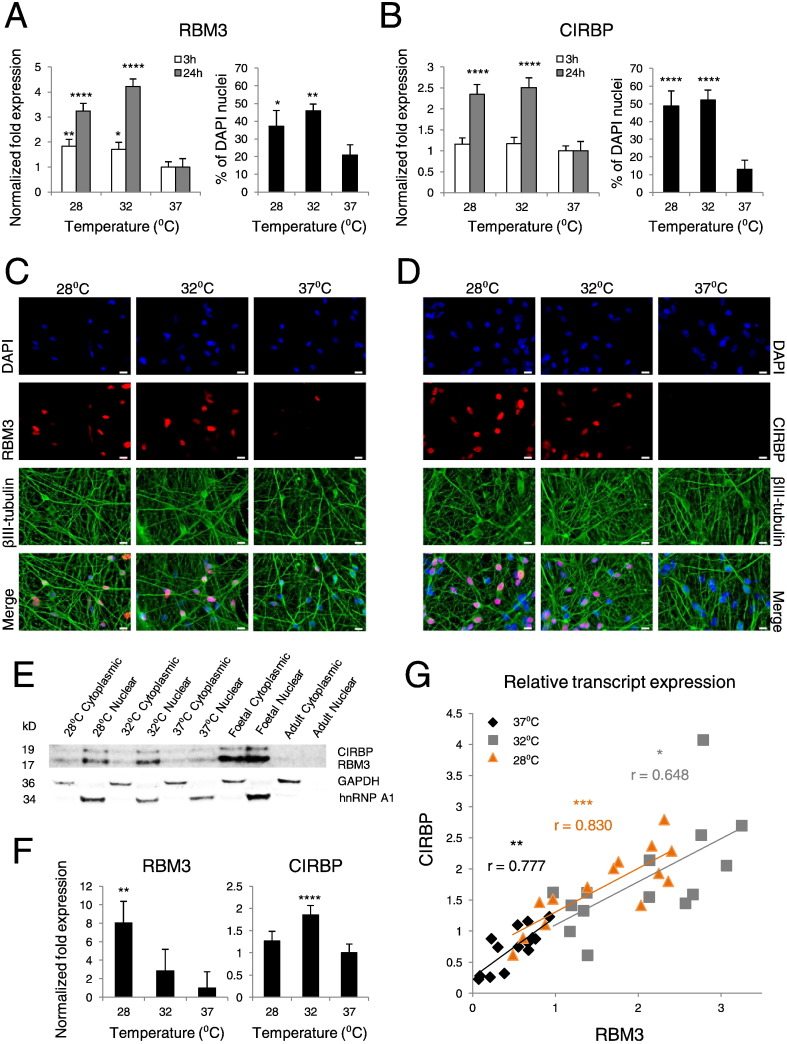
Mild-to-moderate hypothermia elicits a cold-shock response in hCNs. (A) RBM3 transcripts (left, *N* = 3; *n* = 14; *n_HESI_* = 7, *n_HES2_* = 4, *n_IPS1_* = 3) with significant increases after 3 h (32 °C *P* = 0.011, 28 °C *P* = 0.003) and 24 h (*P* < 0.0005). Cell counts for RBM3-positive nuclei (right, *N* = 3; *n* = 6; *n_HES1_* = 4, *n_HES2_* = 1, *n_IPS1_* = 1, mean 37.2% (total 902 out of 2096 cells) at 28 °C, *P* = 0.039; mean 46.0% (total 1050 out of 2377 cells) at 32 °C, *P* = 0.001; mean 20.8% (total 531 out 2062 cells) at 37 °C). For each independent hCN batch and temperature condition, a minimum of 10 fields of view at 63 × were counted (pooled from two replicate coverslips). Counts are presented as mean % + standard error of the mean (SEM). (B) CIRBP transcripts (left, *N* = 3; *n* = 14; *n_HES1_* = 7, *n_HES2_* = 4, *n_IPS1_* = 3, *P* < 0.0005 at 24 h) and cell counts (right, *N* = 3; *n* = 6; *n_HES1_* = 4, *n_HES2_* = 1, *n_IPS1_* = 1, mean 48.9% (total 719 out of 1416 cells) at 28 °C; mean 52.3% (total 985 out of 1882 cells) at 32 °C; mean 13.1% (total 252 out of 1850 cells) at 37 °C, *P* < 0.0005). (C and D) Fluorescent micrographs of hCNs co-stained for neuronal and cold-shock markers, scale bar = 10 μm. (E) Subcellular expression of RBM3 and CIRBP by immunoblot, alongside human foetal and adult cortex. GAPDH and hnRNP A1 are loading controls. The stability of hnRNP A1 expression under mild hypothermic conditions in human cells has reported elsewhere (Danno et al., 1997). (F) Quantitative Western analysis of RBM3 and CIRBP (*N* = 3; *n* ≥ 4; *n_HES1_* ≥ 2; *n_HES2_* = 1, *n_IPS1_* = 1). RBM3 expression was greatest at 28 °C (*P* = 0.002); CIRBP expression peaked at 32 °C (*P* < 0.0005). (G) Correlation of RBM3 and CIRBP transcripts (37 °C *P* = 0.001, 32 °C *P* = 0.012, 28 °C *P* < 0.0005). See also Fig. S1.

To confirm the utility of hCNs to study cryobiological phenomena – and noting that hallmark cold-shock protein induction has not previously been reported in human neurons – we first tested their capacity to elicit this response at mild-to-moderate hypothermic temperatures (Danno et al., 1997, Fujita, 1999, Chip et al., 2011, Yenari and Han, 2012, Tong et al., 2013, Peretti et al., 2015). Within 24 h of cooling, RBM3 and CIRBP transcripts were both increased in hCNs at 28 °C and 32 °C relative to 37 °C, with a concomitant increase in the proportion of RBM3- and CIRBP-positive cells (Fig. 1A and B). RBM3 displayed a more acute and robust response to hypothermia than CIRBP, and both proteins exhibited a predominantly nuclear expression pattern (Fig. 1C and D). Biochemical analysis confirmed upregulation of these chaperones in response to cooling (Fig. 1E and F), and correlation of RBM3 and CIRBP transcript levels at each temperature was supportive of their co-regulation under hypothermic conditions (Fig. 1G). In addition, hypothermic hCNs exhibited a time- and temperature-dependent induction of immediate early transcripts c-Fos (FOS) and c-Jun (JUN) (Yenari and Han, 2012) (Fig. S1 available online). Together, these findings are consistent with a physiological cold-shock response in hCNs.

### 3.2 Cooling Induces ER Stress in Human Neurons with Activation of All UPR Branches

**Fig. 2:**
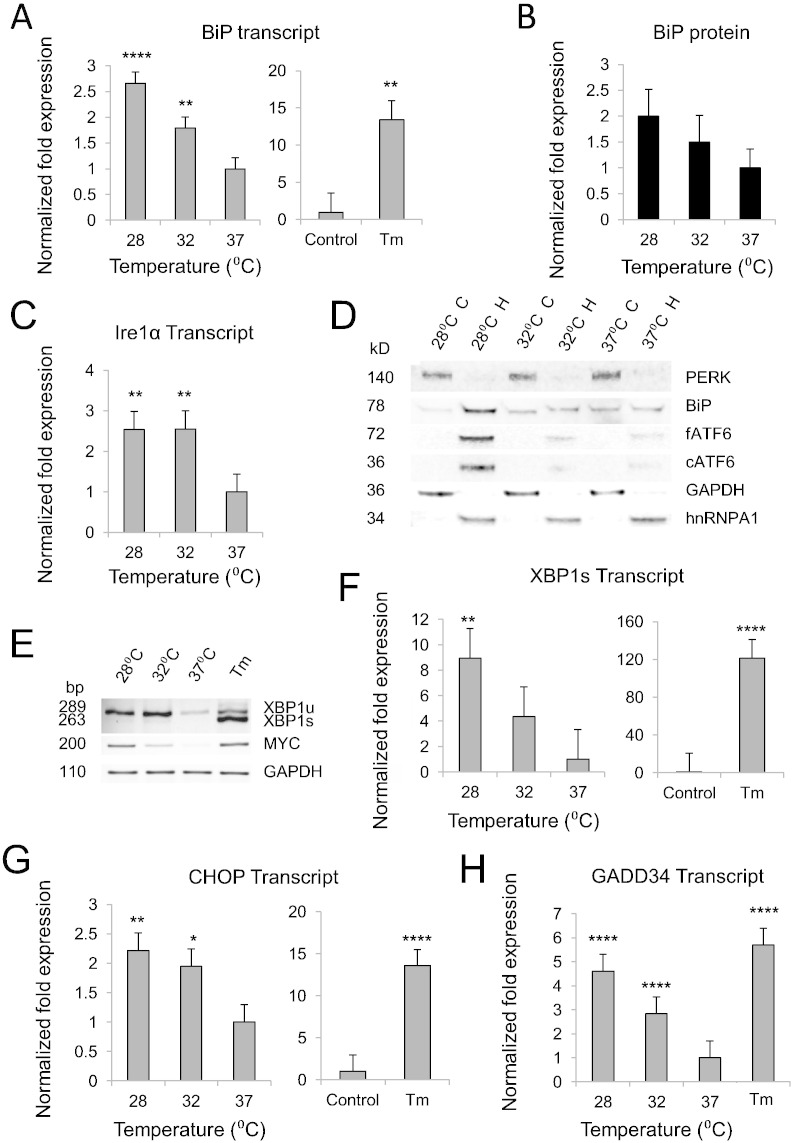
Hypothermia induces mild ER stress in hCNs with full activation of the UPR. (A) BiP transcripts after cooling (left, *N* = 3; *n* = 22; *n_HES1_* = 11, *n_HES2_* = 6, *n_IPS1_* = 5; 32 °C, *P* = 0.006; 28 °C, *P* < 0.0005) or Tm treatment (right, *N* = 3; *n* = 8; *n_HES1_* = 4, *n_HES2_* = 2, *n_IPS1_* = 2, *P* = 0.004). (B) Total BiP protein expression (*N* = 2; *n* = 3; *n_HES1_* = 2; *n_HES2_* = 1; 28 °C, *P* = 0.051). (C) IRE1α transcripts (*P* < 0.01, *N* = 3; *n* = 14; *n_HES1_* = 7, *n_HES2_* = 4, *n_IPS1_* = 3). (D) Immunoblots of fractionated lysates (C = cytoplasmic, H = high-detergent) from hCNs. Note increased BiP, full length (fATF6), and cleaved (cATF6) sitting in the high detergent fraction at 28 °C. This is consistent with nuclear translocation of cATF6 and upregulation of its target transcripts (BiP and unspliced XBP1 — as shown in Figs. 2A, E, and S2B). (E) Gel images of RT-PCR products. Faint bands at 263 bp confirm mild splicing of XBP1 in hypothermic hCNs relative to negative (37 °C) and positive (Tm-treated) controls. GAPDH = reference target. (F) qRT-PCR analysis of XBP1s transcript after cooling (left, *N* = 3; *n* = 22; *n_HES1_* = 11, *n_HES2_* = 6, *n_IPS1_* = 5; 28 °C, *P* = 0.003) or Tm-treatment (right, *N* = 3; *n* = 8; *n_HES1_* = 4, *n_HES2_* = 2, *n_IPS1_* = 2, *P* < 0.0005). (G) CHOP transcripts after cooling (left, *N* = 3; *n* = 22; *n_HES1_* = 11, *n_HES2_* = 6, *n_IPS1_* = 5; 32 °C, *P* = 0.011; 28 °C, *P* = 0.001) or Tm treatment (right, *N* = 3; *n* = 8; *n_HES1_* = 4, *n_HES2_* = 2, *n_IPS1_* = 2, *P* < 0.0005). (H) GADD34 transcripts (*N* = 3; *n* = 7; *n_HES1_* = 3, *n_HES2_* = 2, *n_IPS1_* = 2, 32 °C, *P* < 0.0005; 28 °C, *P* < 0.0005; Tm, *P* < 0.0005). See also Fig. S2.

Since cold-shock can activate PERK (Hofman et al., 2012) and JUN (Fig. S1), both of which are components of the UPR, we postulated that cooling could trigger ER stress — the principal driver of UPR activity (Walter and Ron, 2011). We tested this using Tm as a positive control (Lin et al., 2007). BiP and 94 kDa glucose-regulated protein (GRP94) are key chaperones that respond to ER stress and regulate protein folding (Walter and Ron, 2011). We observed a temperature-dependent induction of BiP transcript, at an order of magnitude less than that produced by Tm (Fig. 2A), whilst GRP94 transcript was elevated at 24 h only in response to Tm treatment (Fig. S2A). BiP protein also showed an increasing trend with cooling (Fig. 2B and D). ER stress initiates a tripartite signalling cascade via 3 ER membrane-associated signal transducers (Walter and Ron, 2011). Once activated by autophosphorylation, the first of these transducers (inositol requiring enzyme 1α (Ire1α)) directs non-conventional splicing of its downstream target, x-box binding protein-1 (XBP1) (Lin et al., 2007). Spliced XBP1 (XBP1s) regulates transcription of several UPR target genes including chaperones and ER-associated degradation (ERAD) components which serve to alleviate ER stress (Hetz and Mollereau, 2014). We found a significant upregulation of total XBP1 and Ire1α (ERN1) transcripts at 28 and 32 °C relative to 37 °C (Figs. 2C and S2B). We also observed an increase in XBP1s transcript after cooling, again to a lesser extent than that produced by Tm (Fig. 2E and F). A second UPR pathway involving cleavage of ATF6 was induced at 28 °C (Hetz and Mollereau, 2014) (Fig. 2D). ATF6 activates transcription of ERAD genes and XBP1 (Hetz and Mollereau, 2014). Within the third pathway, total PERK expression decreased after 24 h cooling and eIF2α was inactive at this time point according to biochemical analysis of its phosphorylated form (Fig. S2C) (Rutkowski et al., 2006). There was however a significant increase in their downstream targets, activating transcription factor 4 (ATF4), DNA damage-inducible transcript 3 (DDIT3, or CHOP) and growth arrest and DNA damage 34 (GADD34) at hypothermic temperatures (Figs. 2G, H, and S2E). In summary, these findings demonstrate a mild ER stress in cooled hCNs, sufficient to activate all branches of the UPR. To our knowledge, this is the first description of a full UPR cascade in cells under hypothermic conditions.

### 3.3 Hypothermic Preconditioning of Human Neurons Requires UPR-Driven ER-Hormesis

**Fig. 3:**
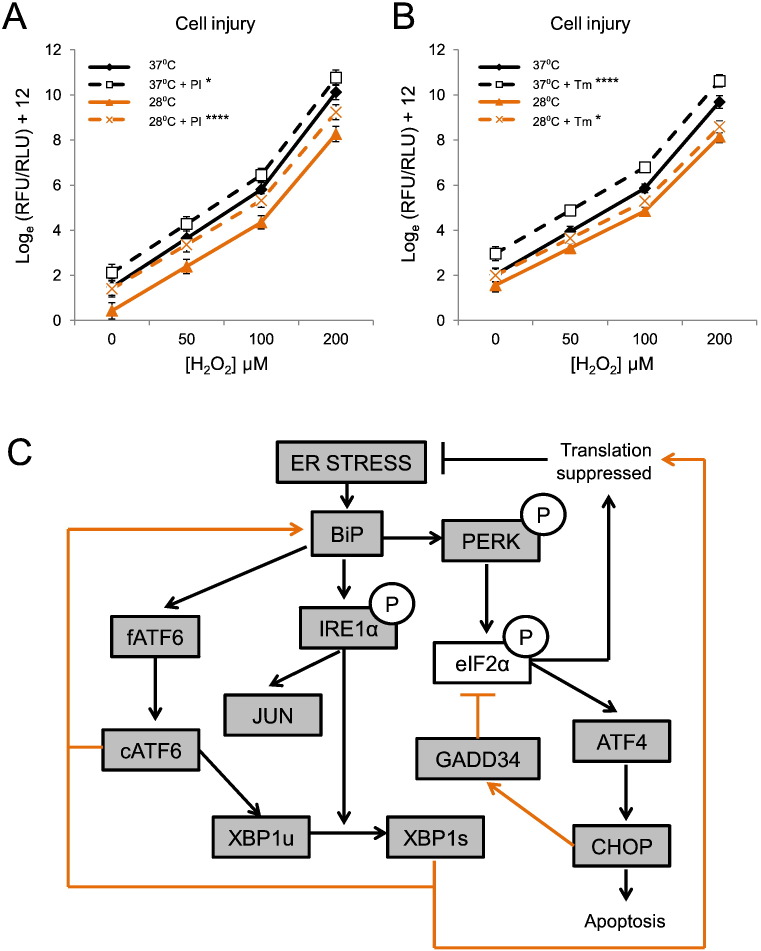
Hypothermic UPR-mediated preconditioning of the ER protects hCNs against oxidative stress. (A) Oxidative stress-mediated injury is increased by PERK inhibitor (PI) (*N* = 3; *n* = 3; *n_HES1_* = 1, *n_HES2_* = 1, *n_IPS1_* = 1; 28 °C, *P* < 0.0005; 37 °C, *P* = 0.016). Hypothermia remained protective only at 200 μM H_2_O_2_ (*P* = 0.023). (B) H_2_O_2_ injury is increased by Tm (*N* = 3; *n* = 7; *n_HES1_* = 5, *n_HES2_* = 1, *n_IPS1_* = 1; 28 °C, *P* < 0.05; 37 °C, *P* < 0.0005). Hypothermia reduced the toxic effect of Tm (*P* = 0.062). Note that log scale was required to accommodate magnitude of injury changes across H_2_O_2_ concentrations in Fig. 3A and B. (C) Proposed mechanism of ER-hormesis in cooled hCNs. UPR pathways are depicted, together with known regulatory feedback pathways. Filled boxes denote components induced at transcript and/or protein level in hCNs with cooling. Phospho-IRE1α and phospho-PERK were not assessed. Orange arrows indicate hormetic elements that resolve the UPR and increase ER resilience to stress. See also Fig. S3.

Mild ER stress with UPR activation inhibits apoptosis (Rutkowski et al., 2006) and pre-conditions neurons to resist more stressful insults — an effect termed ER-hormesis (Mendes et al., 2009, Fouillet et al., 2012). To determine whether ER preconditioning contributes to hypothermic protection of hCNs we chemically modified the ER-UPR cascade during the pre-cooling phase, prior to inducing a standard oxidative stress protocol. PERK inhibitor was used to block the third UPR pathway, whilst Tm was added to induce ER stress (Lin et al., 2007). First we determined dose response curves for each compound in normothermic hCNs to identify concentrations that were non-toxic at baseline (Fig. S3). Multiplexed injury analysis (Materials and Methods) was then applied to hCNs exposed to increasing concentrations of H_2_O_2_, after pre-incubation at 28 °C or 37 °C, with or without PERK inhibitor or Tm. As expected, moderate hypothermia was protective of hCNs (Fig. 3A and B). However, PERK inhibition increased hCN injury at each temperature, abrogating the protective effect of cooling at all but the highest concentration of H_2_O_2_ (Fig. 3A). Tm exacerbated oxidative stress-mediated injury at 37 °C, but this effect was attenuated by pre-conditioning at 28 °C (Fig. 3B), thus directly demonstrating cooling-mediated ER-hormesis. These results confirm that full hypothermic neuroprotection requires an intact UPR to prime the ER against intensively toxic insults. The influence of cooling on this proteostatic cascade in hCNs is summarized in Fig. 3C.

## 4 Discussion

In acute injury, mildly enhancing the UPR can rescue neurons from programmed cell death and instigate adaptive ER preconditioning (Hetz and Mollereau, 2014). In hCNs, PERK activity was essential for hypothermic preconditioning against an oxidative challenge. Mild XBP1 splicing after 24 h of cooling, together with a substantial increase in unspliced XBP1 mRNA (Figs. 2E, F, and S2B) indicate that Ire1α and ATF6 were active within the cooling period (Hetz and Mollereau, 2014). Moreover, the increase in BiP transcript after 24 h is consistent with prior activation of ATF6 and splicing of XBP1 (Hetz and Mollereau, 2014). Enhanced injury with PERK inhibition at 37 °C may reflect a constitutive proteostatic function of the UPR in long-term culture — potentially through buffering oxidative processes (Cullinan et al., 2003). It might also explain why hypothermic induction of some PERK branch-specific components was not observed; eIF2α phosphorylation does occur under deep hypothermic conditions (10 °C) and contributes to the global suppression of protein translation in mammalian cell lines (Roobol et al., 2009, Hofman et al., 2012), but it may be undetectable biochemically in the context of mild ER stress (Rutkowski et al., 2006). Equally, since PERK-mediated translational repression is subject to homeostatic autoregulation by phosphatases (Lin et al., 2007), a resolving influence of cooling on eIF2α activation is supported by hypothermic induction of GADD34 in hCNs (Figs. 2H and 3C) (Ma and Hendershot, 2003). In this respect, rather than signifying the duration limit of protective cooling, the CHOP induction observed would be a pre-requisite for GADD34-mediated negative feedback on eIF2α (Halterman et al., 2010). Accordingly, others have highlighted the protective role of CHOP in neuronal systems (Chen et al., 2012, Engel et al., 2013). The fact that 24 h cooling did not increase levels of the pro-apoptotic marker Bax (Fig. S2E) is in line with previous studies (Yenari et al., 2002) and further supports our conclusion that this duration and depth of hypothermia produced an adaptive UPR. Potentially, cold-shock proteins may complement this cascade by relieving translational repression of critical mRNAs (Peretti et al., 2015), and limiting CHOP-mediated apoptosis (Saito et al., 2010).

During an adaptive stress response UPR branches undergo complex homeostatic self-regulation (Fig. 3C). Thus the cross-sectional UPR profile captured in our hCN system cannot convey the dynamic nature of these pathways. However, our analysis at 24 h intimately links UPR activation to neuronal preconditioning, since this was the point at which H_2_O_2_ was applied, and it further indicates co-ordination of ER-hormesis with cold-shock protein induction. The lack of GRP94 induction after 24 h cooling may reflect the half-life of its transcript or the selective nature of this chaperone, whose client list is smaller than that of BiP — in particular, GRP94 is not induced at high temperatures (Marzec et al., 2012). Furthermore, prolonged ER stress leads to sequential activation then deactivation of Ire1α, ATF6 and PERK pathways respectively — which might explain the bias of UPR components towards the PERK arm at 24 h (Tabas and Ron, 2011). Nevertheless, our transcript analysis revealed distinct patterns of UPR responses resulting from two different stresses; whilst BiP, GRP94, XBP1s and CHOP dramatically increased with Tm, ATF4 and GADD34 induction were comparable between Tm and cooling. Therefore, in contrast to models described elsewhere (Rutkowski et al., 2006), the negative regulation of eIF2α appears to take precedence over unloading the ER in cooled human neurons. This relief of translational repression may confer tolerance to a prolonged hypothermic state (Peretti et al., 2015, Moreno et al., 2013).

In the clinic, ‘preconditioning’ is typically ascribed to a transient mild stress followed by a recovery interval (Nishio et al., 2000, Stetler et al., 2014). Here we have applied this term in its broadest sense — i.e., a subtoxic cellular stress that can lead to a protective state (Stetler et al., 2014) in order to account for the proteostatic priming observed during our pre-incubation phase of cooling. This definition circumvents the need for a re-warming phase which would confound analysis of oxidative injury by inducing relative hyperthermic and hypoxic stresses (Liu et al., 1994, Lleonart, 2010, Chip et al., 2011, Neutelings et al., 2013). Hypothermic preconditioning may reconcile conflicting data describing UPR modulation in neuronal health; (1) that ER stress can elicit UPR-mediated hormesis (Mendes et al., 2009, Fouillet et al., 2012), (2) that circumventing UPR-mediated translational repression promotes long-term survival (Moreno et al., 2013), and (3) that inhibiting eIF2α phosphatases resolves ER stress (Kiskinis et al., 2014). This highlights the importance of fine-tuning the entire network, rather than adjusting a single pathway or component — such a combinatorial approach has been proposed for amyotrophic lateral sclerosis (Kiskinis et al., 2014). Whilst hypothermic preconditioning originates from the acute injury setting, impaired stress responses underlie several neurodegenerative disorders (Hetz and Mollereau, 2014) and preconditioning in general is a proposed target (Stetler et al., 2014). Cooling has recently demonstrated some benefit in an in vivo model of spastic paraplegia (Baxter et al., 2014) and Peretti et al. (2015) observed that neurodegenerative synaptic loss could be partially rescued through early cooling-induced enhancement of RBM3 expression. Conceivably, this temporal dependency might relate to hypothermia-mediated proteostatic priming, elicited prior to the build-up of a significant protein aggregate load. Whether the hypothermic preconditioning described here is linked to a cytoprotective mechanism that is synergistic with the preservation of synaptic plasticity is worthy of further investigation (Peretti et al., 2015). Ultimately, disease stage and neuronal subtype would determine whether enhanced or prophylactic preconditioning could be useful in the context of neurodegeneration (Saxena et al., 2009).

In response to cooling, hCNs displayed all the hallmarks of an adaptive, preconditioning UPR response: mild ER stress and activation of all 3 ER-stress transducers, a low level of CHOP induction that was insufficient to effect apoptosis, absence of detectable levels of phospho-eIF2α, and residual expression of key ER chaperones (Rutkowski et al., 2006, Tabas and Ron, 2011). The reversibility of these effects and the period over which they would remain protective is currently unknown and is part of ongoing work. Since our cooling paradigm can be used to titrate UPR activation, it represents a simple method to address subtle but important effects dictating adaptive versus maladaptive outcomes of this cascade in any cell type. We propose that ER-hormesis is an important outcome of the cold-shock response that protects human neurons from both ER and oxidative stress. This ‘cross-tolerance’ effect (Rutkowski et al., 2006, Stetler et al., 2014) places exponential value on the molecular neurobiology of cooling, which may deliver novel therapeutic targets for an unmet need.

## Author Contributions

N.M.R. conceived the research, conducted the literature search, designed and performed the experiments, compiled the figures and wrote the manuscript. P.C. analysed the data. N.M.R., P.C., R.P. and B.T.S. interpreted the results. S.C. supervised the project. All authors contributed to the discussion and the final draft of the paper.

## Role of the Funding Sources

Funding sources did not have any involvement in the study design; the collection, analysis and interpretation of data; writing of the report; or the decision to submit the article for publication. The authors declare no conflicts of interest.
