# Supplementary material for: Hypothermic Preconditioning of Human Cortical Neurons Requires Proteostatic Priming
Source: eBioMedicine. 2015 Apr 11;2(6):528–35. doi: 10.1016/j.ebiom.2015.04.004 (PMC4534756; doi:10.1016/j.ebiom.2015.04.004)
Supplement: Supplementary file 1 — Supplementary material. [file mmc1.pdf]

SUPPLEMENTARY FIGURES

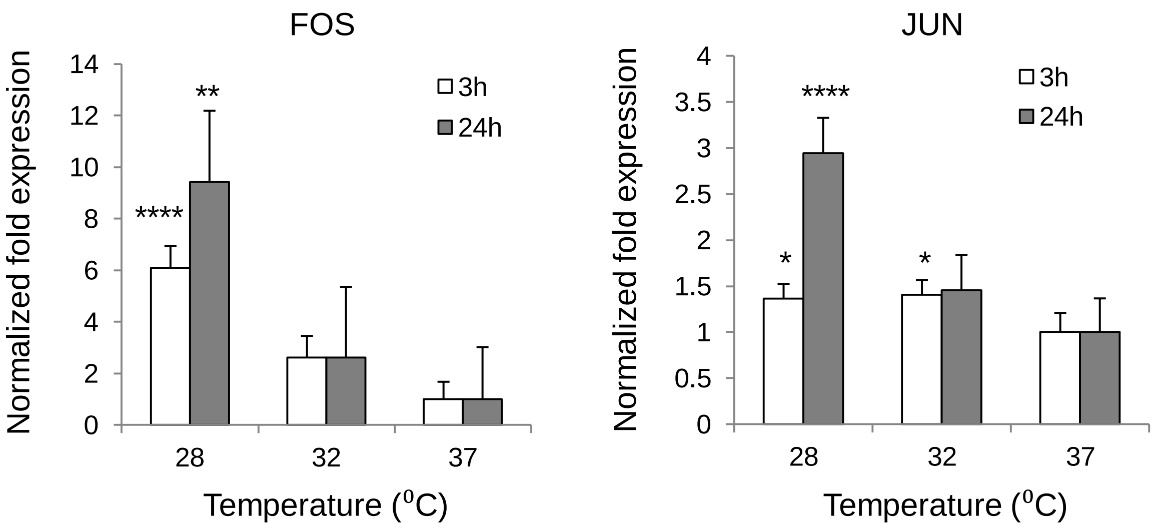

Figure S1. Immediate Early Gene Induction in Cooled hCNs, Related to Figure 1. Immediate early transcripts were analysed by q-RT-PCR (N=3; n=14; n_{HES1}=7, n_{HES2}=4, n_{IPS1}=3). Left; FOS induction at 28^{0}C at 3 h (P<0.0005) and 24 h (P=0.002), right; JUN induction at 3h at 32^{0}C (P=0.01) and 28^{0}C (P=0.021) and at 24 h at 28^{0}C (P<0.0005).

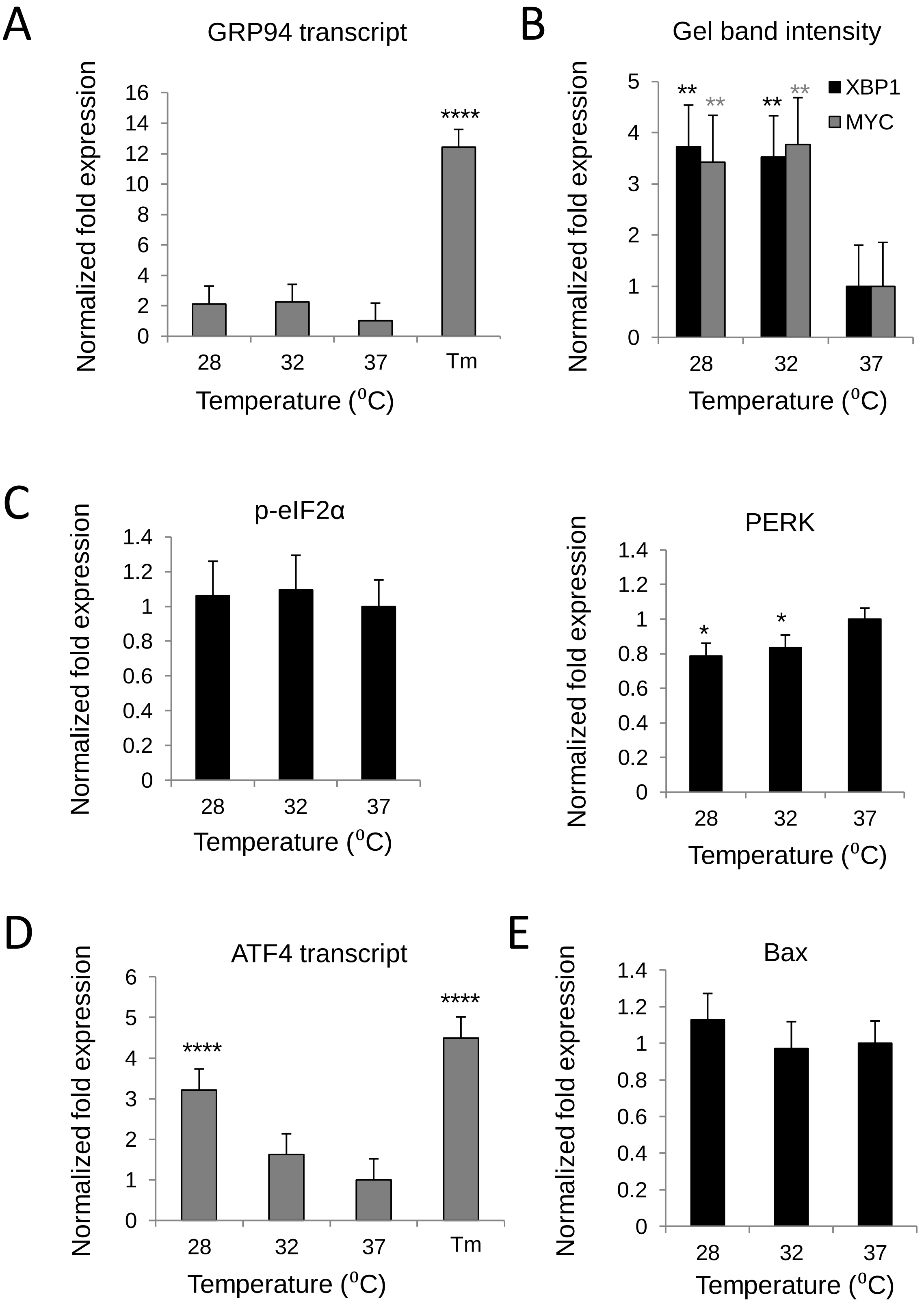
A
GRP94 transcript
Normalized fold expression
0
2
4
6
8
10
12
14
16
****
28
32
37
Tm
Temperature (⁰C)
B
Gel band intensity
Normalized fold expression
0
1
2
3
4
5
**
**
**
**
XBP1
MYC
28
32
37
Temperature (⁰C)
C
p-eIF2α
Normalized fold expression
0
0.2
0.4
0.6
0.8
1
1.2
1.4
28
32
37
Temperature (⁰C)
PERK
Normalized fold expression
0
0.2
0.4
0.6
0.8
1
1.2
1.4
*
*
28
32
37
Temperature (⁰C)
D
ATF4 transcript
Normalized fold expression
0
1
2
3
4
5
6
****
****
28
32
37
Tm
Temperature (⁰C)
E
Bax
Normalized fold expression
0
0.2
0.4
0.6
0.8
1
1.2
1.4
28
32
37
Temperature (⁰C)

Figure S2. Adaptive UPR Activation in Cooled hCNs, Related to Figure 2.

(A) GRP94 transcripts (N=3; n=7; n_{HES1}=3, n_{HES2}=2, n_{IPS1}=2, 32^{0}C, P=0.432; 28^{0}C, P=0.473; Tm, $P<0.0005$).

(B) RT-PCR analysis of transcripts (normalized to GAPDH then normothermic control), with significant increases for MYC (N=2; n=5, n_{HES1}=3, n_{HES2}=2, $P<0.01$) and unspliced XBP1 (XBP1u, N=2; n=7, n_{HES1}=5, n_{HES2}=2, $P<0.01$).

(C) Quantitative Western analysis (N=3; n=5; n_{HES1}=3; n_{HES2}=1, n_{IPS1}=1) of phospho-eIF2α (left) and total PERK (right, $P<0.05$).

(D) ATF4 transcripts (N=3; n=7; n_{HES1}=3, n_{HES2}=2, n_{IPS1}=2, 32^{0}C, P=0.215; 28^{0}C, $P<0.0005$; Tm, $P<0.0005$).

(E) Quantitative Western analysis of apoptotic regulator Bcl-2 associated X protein (Bax) (N=3; n=6; n_{HES1}=4; n_{HES2}=1, n_{IPS1}=1).

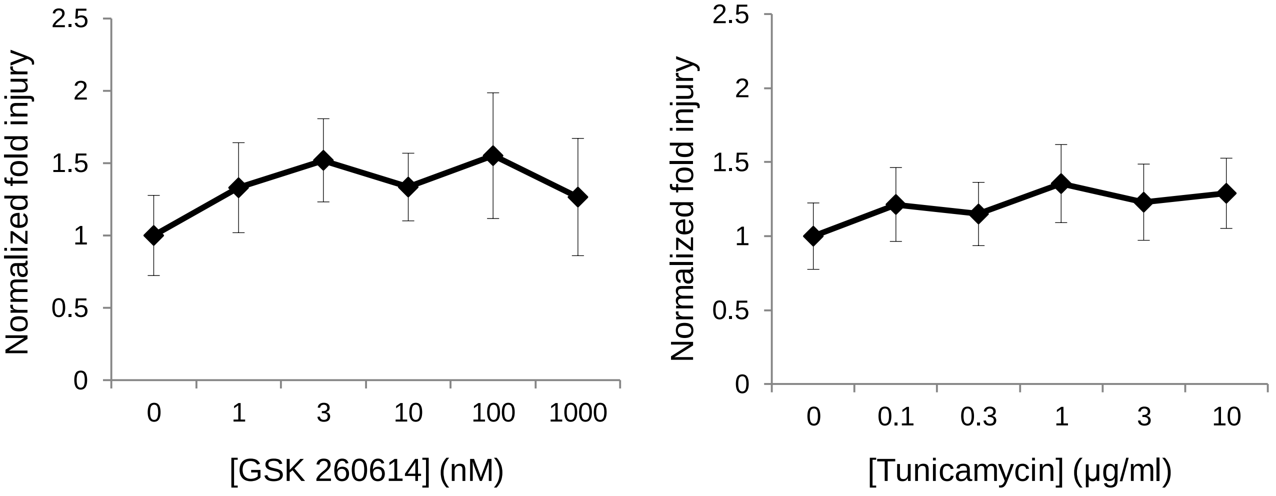

Figure S3. Dose-Response Curves in Normothermic hCNs, Related to Figure 3.

hCNs were treated with compounds as indicated for 24 h at 37^{0}C. Left; PERK inhibitor (GSK 2606414), N=3; n=5; n_{HES1}=2, n_{HES2}=2, n_{IPS1}=1, right; Tm N=3; n=8; n_{HES1}=2, n_{HES2}=3, n_{IPS1}=3. Injury ratio was normalized to untreated control and is presented as mean ± SEM. No significant change in injury was observed over the dose ranges tested for either compound.

SUPPLEMENTARY MATERIALS AND METHODS

Primer Sequences Used for RT-PCR

Gene target	Forward primer	Reverse primer
**ATF4**[a]	TCAAACCTCATGGGTTCTCC	GTGTCATCCAACGTGGTCAG
**BiP (HSPA5)**[b]	CATCACGCCGTCCTATGTCG	CGTCAAAGACCGTGTTCTCG
**CIRBP**	TAGAGGAGGAGGGGACCGAG	TCACTGTAGCCACCACTCTG
**DDIT3**[c]	ACCAAGGGAGAACCAGGAAACG	TCACCATTCGGTCAATCAGAGC
**ERN1**[c]	TGGGTAAAAAGCAGGACATCTGG	GCATAGTCAAAGTAGGTGGCATTCC
**EIF4A2**	GAAGCCTTCCGCTATTCAGCA	CTTGGGTCTCCTTGAACTCAATC
**FOS**[d]	CTACCACTCACCCGCAGACT	AGGTCCGTGCAGAAGTCCT
**GADD34**[a]	CGACTGCAAAGGCGGC	CAGGAAATGGACAGTGACCTTC
**GAPDH**[e]	GAGTCCACTGGCGTCTTCAC	ATGACGAACATGGGGGCAT
**GRP94**[a]	TTGGTGTCGGTTTCTATTCC	GCTGGGTATCGTTGTTGTG
**JUN**	TCGACATGGAGTCCCAGGA	GGCGATTCTCTCCAGCTTCC
**MYC**	CCAGGCTTAGATGTGGCTCT	CTCTGACCTTTTGCCAGGAG
**RBM3**[f]	CTTCAGCAGTTTCGGACCTA	ACCATCCAGAGACTCTCCGT
**XBP1**[g]	TTACGAGAGAAAACTCATGGCC	GGGTCCAAGTTGTCCAGAATGC
**XBP1s**[h]	TGCTGAGTCCGCAGCAGGTG	GCTGGCAGGCTCTGGGGAAG

[a]Li et al., 2015; [b]Harvard Primer Bank; [c]Lin et al., 2007; [d]Bilican et al., 2014; [e]Caradec et al., 2010; [f]Wellmann et al., 2004, [g]Samali et al., 2010, [h]van Schadewijk et al., 2012.

SUPPLEMENTARY REFERENCES

Caradec J., Sirab N., Keumeugni C., Moutereau S., Chimingqi M., Matar C., Revaud D., Bah M., Manivet P., Conti M., et al., ‘Desperate house genes’: the dramatic example of hypoxia, *Br. J. Cancer* **102**, 2010, 1037-1043.

Harvard primer bank (http://pga.mgh.harvard.edu/primerbank/index.html).

Li J., Cai X., Xia Q., Yao K., Chen J., Zhang Y., Naranmandura H., Liu X. and Wu Y., Involvement of endoplasmic reticulum stress in all-trans-retinal-induced retinal pigment epithelium degeneration, *Toxicol. Sci.* **143**, 2015, 196-208.

Wellmann S., Bührer C., Moderegger E., Zelmer A., Kirschner R., Koehne P., Fujita J. and Seeger K., Oxygen-regulated expression of the RNA-binding proteins RBM3 and CIRP by a HIF-1-independent mechanism, *J. Cell Sci.* **117**, 2004, 1785–1794.

Samali A., FitzGerald U., Deegan S. and Gupta S., Methods for monitoring endoplasmic reticulum stress and the unfolded protein response, *Int. J. Cell Biol.* **2010**, 2010, 830307.

van Schadewijk A., van’t Wout E.F.A., Stolk J. and Hiemstra P.S., A quantitative method for detection of spliced X-box binding protein-1 (XBP1) mRNA as a measure of endoplasmic reticulum (ER) stress, *Cell Stress Chaperones* **17**, 2012, 275-279.
